# An ethnographic study exploring the experiences of patients living with cancer illness in support group settings in KwaZulu-Natal, South Africa

**DOI:** 10.4102/phcfm.v13i1.2303

**Published:** 2021-03-15

**Authors:** Nkosinathi Mncwabe, Khumbulani W. Hlongwana, Themba G. Ginindza

**Affiliations:** 1 Discipline of Public Health Medicine, School of Nursing and Public Health, University of KwaZulu-Natal, Durban, South Africa

**Keywords:** support groups, ethnography, qualitative research, purposive sampling, cancer

## Abstract

**Background:**

The major strength of support groups stems from their ability to help patients manage their health within and outside the traditional hospital settings. Despite the known benefits of support groups for people living with cancer, ethnographic studies documenting the cancer patients’ experiences of living with cancer within the support group contexts in KwaZulu-Natal are scarce.

**Aim:**

The aim of this study was to examine the experiences of patients living with cancer within a support group setting.

**Setting:**

The study setting was support groups in KwaZulu- Natal, South Africa.

**Materials and Methods:**

This study was conducted using, participant observation, focus group discussions and in-depth interviews. Data were generated over a 3-month period. Purposive sampling was used to identify the information-rich participants. Thematic data analysis was performed in order to obtain insights into the collective meaning of data generated.

**Results:**

Participants viewed the support group settings as creating an environment with a unique sense of community. This was in contrast with the sense of isolation, rejection and lack of empowering knowledge on cancer, often experienced outside these contexts. Moreover, the support groups were lauded for facilitating positive relationships with family and friends and providing a safe space for members to freely express their emotions.

**Conclusion:**

Psychosocial support provided by support groups can help to ameliorate the distress caused by cancer diagnosis and its treatment; however, these support groups are still few and far in between. Therefore, there should be a greater investment in establishing support groups.

## Background

Despite cancer being a preventable and curable disease, it remains the most devastating disease in the world today, particularly in the sub-Saharan Africa (SSA), where the disease is often diagnosed late, resulting in poor treatment outcomes.^1^ Most of the research conducted in Africa aimed at tackling the cancer epidemic in the continent and focused largely on curative medical treatment interventions. Less emphasis has been placed on the provision of psychosocial care and support for those affected by cancer.^2^ The literature suggests that support groups are an effective intervention strategy for cancer care and support.^3^ Scientific evidence from the past decade shows that support groups are the most common social intervention strategy used to deal with a variety of emotional consequences of cancer.^3^ Similarly, support groups have been lauded for providing high-quality care to patients with life-threatening illnesses using human rights.^4^ Despite the value of support groups in providing psychosocial care to patients and their families in South Africa, this intervention has to navigate or even mediate a country’s polarised healthcare system.

The healthcare services in South Africa are characterised by two parallel systems, namely, public and private healthcare,^5^ with the most patients using the public healthcare system,^6^ given that the majority of South Africans are unable to afford the profit-driven private healthcare. Currently, the literature in South Africa is biased towards the studies focusing on the curative treatment interventions,^7^ negating the documentation of the patients’ daily ethnographic experiences of living with the disease. A case in point is the study that used survey tools to investigate the biomedical dimensions of coping with cancer.^8^ Such studies have involved either post-discharged patients or outpatients using surveys and may have missed important data on experiences of patients living with and/or undergoing cancer treatments, either within or outside the hospital settings. As a result, researchers and other interventionists have become more aware of situational and psychological responses, as well as social factors, influencing the quality of life of cancer patients, and hence, this resulted in the establishment of support groups and palliative care centres.^9^ The positive effects of support groups, such as increasing long-term coping skills,^10^ decreasing emotional stress^11^ and improving quality of life,^10^ are well documented. However, these studies are rarely conducted through qualitative methods, and hence, the understanding of how patients living with cancer experience the illness may not be of sufficient depth.

In South Africa, informal support groups and palliative care centres are developing as popular support structures for assisting people with chronic diseases, including for cancer and human immunodeficiency virus (HIV).^10^ This is partly because of the need for professional help and psychosocial intervention aimed at assisting the large number of people diagnosed with these diseases.^10^ However, in our review of literature, sourced using PubMed, Science Direct and World Health Organization search engines, we could not find any study investigating experiences and perceived benefits of participating in support groups in KwaZulu-Natal, South Africa.^12^ In this article, we document the experiences of patients living with cancer in support group settings, including the cultural construction of the illness, using ethnographic tools.

## Materials and methods

### Study setting

This study was conducted on the patients living with cancer and attending two cancer support groups, located in Pietermaritzburg’s townships of Imbali and Sobantu, respectively. As part of the overall study objectives, the Imbali and Sobantu support groups were established from scratch and reconfigured from the existing HIV and acquired immunodeficiency syndrome (AIDS) support group, respectively. The reconfiguration of an existing support group in Sobantu to include patients with cancer was informed by an increasing number of people living with comorbid HIV and AIDS, and cancer in the area. Incorporating cancer aspects into this support group was deemed to be necessary in order to cater for the needs of this emerging group. The leader of the 11 member Sobantu support group had the comorbid HIV-related illness and AIDS and cancer. However, there was no support group in Imbali township, and therefore, the support group was formed by the lead researcher from the scratch, which had 12 members at the time of data collection.

### Study design

This study explored patients’ experiences of living with cancer within the support group settings in the Imbali and Sobantu townships in the Msunduzi Municipality using an ethnographic study design. This design has proven effective in the studies aimed at understanding how the secluded and subdued communities experience a particular phenomenon.^13^ This design was appropriate for this study, given its ability to generate rich data captured in subtle nuances through the researcher’s immersion in the research setting.^13^

### Study population

The study population consisted of adult patients (over the age of 18 years) living with cancer or cancer survivors attending the support group. To be a member of the support group, a person should be living with cancer and other illnesses, supporting someone living with cancer or a cancer survivor. The establishment or reconfiguration of these support groups was part of the broader Multinational Lung Cancer Control Programme (MLCCP) objectives. The MLCCP is a multinational programme involving four countries, namely, Eswatini, Tanzania, Kenya and South Africa, however; this project was conducted in South Africa and KwaZulu-Natal in particular. The establishment and reconfiguration of support groups were achieved through the assistance of local community health workers and a social worker from Cancer Association of South Africa.

### Support groups and their operations

Sobantu and Imbali support groups included the membership of 11 and 12 people, respectively. Support group meetings were held weekly for 1-2 h, with the attendance varying between 8 and 12 members per meeting. The reasons for this variation in numbers for weekly attendance included hospital appointments, government grant payouts, inclement weather and being too sick or changing the residential area. Both Sobantu and Imbali support groups consisted of recently diagnosed cancer patients, patients in remission, cancer survivors or carers of cancer patients. At the beginning of each meeting, the researcher and social worker introduced themselves to the group in order to accommodate any new member, as groups were continuously growing. The purposes of each meeting for the day were explained. The support group sessions included the following topics:

1. Introduction and orientation.
2. Cancer and access to treatment.
3. The emotional experience of having cancer.
4. Cancer, disclosure and stigma.
5. Coping, problem solving and stress management.
6. Cancer in the household, human rights and stigma.

The above-mentioned topics were decided by the group members, the facilitator, as well as the social worker.

### Sample size determination and sampling strategy

The support group sample size was 28, and the support group members included 23 participants. The membership criteria were participants who had attended at least three support group meetings, and 16 participants were selected for in-depth interviews from the main sample size. The 16 participants were purposively selected for inclusion in the study, provided they were willing to participate, in addition to being physically and emotionally fit at the time of data collection.

### Ethical considerations

This study was approved by the Biomedical Research Ethics Committee of the University of KwaZulu-Natal (BE555/18). Verbal permissions were further sought from the concerned authorities, including the counsellors in each of the two sites, who had been taken through the purpose and the objectives of the project. All potential participants provided written informed consent prior to participating in the research aspects of the support groups. Participation of all respondents in the study was strictly voluntary, and anonymity was maintained to protect the participants’ identities. All participants were encouraged to maintain respect, dignity and freedom of speech for everyone participating in the study.^14^

### Data generation

Data were generated through in-depth interviews, focus group discussions, journaling and observations over a period of 3 months (November 2018 to February 2019). Interviews were conducted by the lead researcher in both English and IsiZulu depending on the participant’s preferred language. Field notes were written in 14 support group meetings (seven in each support group site), covering six sessional topics listed in the previous section. Interviews conducted in IsiZulu were transcribed verbatim before being translated into English. Individuals within these two support groups were invited to participate in the focus group discussions. In total, four focus group discussions were conducted, with each group having eight members. The focus group discussions were conducted in the same venues where these support group meetings were held. The central theme in the focus group discussions was the patients’ experiences of living with cancer.

### Data entry and analysis

This research employed two data entry systems, namely, field notes and journaling. For field notes data entry system, the data were captured onsite and within the support groups, and further refined and expanded once the researcher was in the office. This allowed for quick resolution of data omissions, errors and inconsistencies. Likewise, journaling or keeping personal diaries was used as an appropriate data entry method during data generation. Personal diaries were maintained by support group members and were used for reflection purposes. The transcriptions were read by the researcher to ascertain the major theme emerging and to develop a coding frame. The researcher made detailed notes on the interview transcript regarding the content and theme.

Thematic data analysis was applied to get insights on the collective meaning of data generated. All the emergent themes were supported by the data and not imposed by the researcher.

## Results

The educational status of participants varied widely, with 24.9% having no formal education, whilst 43% and 57% of the participants were men and women, respectively. Of the 57.2% participants who had completed high school, 21% and 29% were men and women, respectively. Participants with post-matric qualification constituted 17.9%, with the majority of them being women (65%). The majority (82.1%) of participants were unemployed and relied on government grants for their livelihoods (Table 1).

**TABLE 1:** Participants’ demographic profiles.

Characteristics	Number	%
**Age (years)**		
30–39	4	14.3
40–49	13	46.4
50–59	11	39.3
**Gender**		
Men	5	17.9
Women	23	82.1
**Employment status**		
Employed	5	17.9
Unemployed	23	82.1
**Educational status**		
Primary	12	42.8
Secondary	11	39.3
Tertiary	5	17.9

Four themes emerged from the data analysis: (1) patients’ conceptualisation of cancer as a fatal disease, (2) patients’ reflections on their treatment by medical staff, (3) stigma and disclosure complexities and (4) rationalising the illness through the lenses of faith and spirituality. These themes helped not only to understand patients’ experiences of living with cancer but also the cultural construction of their illness. We learned that these findings carry profound meanings within the local context and thus provide insights into our understanding of the meaning of illness from the perspectives of those enduring the suffering.

### Patients’ conceptualisation of cancer as a fatal disease

*Ask God two things: if he can’t cure you, he must take you away! – R1*.

There was a convergence of opinions in participants’ perceptions of diagnosis with cancer, as a distressful emotional experience. This distressful emotional experience manifested through the use of the words, such as shock (R2 and R3), fear (R2, R4 and R7), anger (R3 and R5), devastation and hopelessness (R4, R5 and R6). Participants recognised the whole cancer care continuum from diagnosis to treatment as an emotionally challenging experience (R1 and R7). Apart from the structural conditions of community areas, the participants’ experiences of illness were affected by the wider social and familial circumstances:

‘I was horrified, terrified, shocked, traumatized, call it what you want to name it, cried myself to sleep for three months in a row, worried how am I going to tell the rest of my family. I asked myself, how will society look at me, there are a lot of things so basically, it just horrible, terrible, traumatic, mind boggling.’ (R2, active society member, recovering cancer patient, 06 December 2018)

‘The thought of cancer, it is such a terrifying thought, the disease, it disrupts you, it wants to kill you that is how I was feeling the other day, you saw me. I was in so much pain and stress but thank God. I tried to overcome it, prayer is helping a bit, as you can see, so is meditation.’ (R6, recovering cancer patient, retired factory worker, 03 December 2018)

‘Ok TB is something you can live with right, however cancer on the other hand is deadly, so I feel that it should be separated from other diseases.’ (R3, patient, retired engineer and community activist, 21 November 2018)

‘Cancer is a killer, it blocks you, it brings fatigue, makes you skinny, purely cancer is witchcraft.’ (R4, cancer patient, housewife, 11 February 2019)

‘As a cancer patient, you feel like I can overcome this, I have got to put up with the battle. Fight, you have got to fight for your life, it’s a life and death struggle.’ (R5, cancer patient, support group member, 29 November 2018)

Participants had to strike a balance between wanting to know the implications of their diagnosis (R6 and R7), whilst also trying other alternative therapies. In most cases, patients who were newly diagnosed experienced the feelings of shame and guilt for developing cancer and at times suicide ideation (R6 and R7):

‘I fight with God every day, you know, when I pray, I ask God, “Why must it be me?” I used to say to God, “But God I mustn’t be like this.” You know sometimes, when I am down, I am being honest now, I say “let me take this cellphone wire and put it on my neck” and say “it’s over.” Then I say “no, that’s like I am a coward”.’ (R6, recovering cancer patient, retired factory worker, 03 December 2018)

‘When the pain was getting so much, such that I couldn’t take it, I said God rather take me away.’ (R7, active support group member, self-employed, 04 December 2018)

Likewise, patients’ relatives also expressed feelings of distress in their support roles.

Participants shared converging views about not receiving any follow-up services at the primary care centres, as they felt that they were not provided with any options on how to deal with a deadly disease (R1 and R7). For example, a wheelchair-bound patient suffering from stomach cancer (R1) shared her difficult experiences:

‘And the doctors said they can’t do anything because I would be like a vegetable and that used to make me sicker. That is when I picked up the hypertension because I used to cry when the doctors told me. I couldn’t move myself, and I used to lay in the bed and cry to God, but why? What have I done wrong? You know I was a person that used to help people, I used to help people and fight for their salaries, Why now me? Where did I go wrong?’ (R1, palliative care patient, unemployed, 29 November 2018)

As in the case of R7, accepting these physical limitations was often difficult:

‘Definitely as a patient, you feel that I am a patient, I can overcome this, but you get all these thoughts in your mind but at the end of the day you’ve got to put up battle, fight, you’ve got to fight for your life, it’s a life and death struggle.’ (R7, active support group member, self-employed, 04 December 2018)

The sentiments from R7 reveal patient’s vulnerability and cry for support and care. Whilst some participants were fortunate enough to have support structures, others were not. As a result, the support group network was the only source of support, which provided members of the support group with a strong sense of community. According to R12, support groups provided a safe, trusting and caring space in contrast to the type of care from family members. Attesting to this assertion is the fact that members of the support groups shared some issues that had not been shared with their family members:

‘Since I started attending this group, I feel like I have a new family, a cancer family, a supportive family.’ (R11, support group member, self-employed, 03 December 2018)

‘I never had a family, I do not know of a loving family, my own family never loved me. I thank God for connecting me with such a group.’ (R14, active support group member, 06 December 2018)

### Patients’ reflections on their treatment by medical staff

The quality of interaction between the healthcare professionals and patients living with cancer plays an important role in how the care is provided and received from the diagnosis to treatment. For example, the results of this study revealed that patients generally felt rejected and unembraced by the healthcare system, as they considered the health workers to be unhelpful and rude (R8 and R9). This was combined by a lack of psychosocial programmes catering for patients’ needs (R5, R8 and R9):

‘I go to Dr. X and Dr. Y, so my file is what follows and not my personal relationship with the doctor, there is no personal relationship with the doctor you understand. You are reduced to a number, because let me tell you something Nkosinathi [*Researcher*], I picked up the phone one day wanting to make an appointment with Hospital X. I called and said my KZN number is this because my actual name does not exist, but I am just a number.’ (R8, support group member, runs a community crèche, 06 December 2018)

‘One day when I went to do one of my chemos, I had to wait from the morning until the evening to be attended by a doctor. Luckily, one of nursing sisters noticed and asked the other nurses why I hadn’t been seen. The nurse replied, “I am going for lunch.” At this moment, I got very upset and told them I left my house very early, even before having breakfast, I need to be attended to.’ (R9, active support group member, pensioner, 11 February 2019)

There were also isolated cases of positive stories of the patients’ relationship with healthcare professionals, as in the case of R1:

‘And then, there was a black doctor, and then he said I am happy in my heart now that I could help you to find what is wrong. Because if I went home, he would feel that he never helped a patient. She was very nice you know. And even all the oncologists are very nice. And uhm, my oncologist, she is so loving, the moment she sees a patient, she just wants to grab them, you know she is like happy to see them.’ (R1, palliative care patient, unemployed, 29 November 2018)

Patients were also concerned about the lack of resources, long waiting lines and unsuitable treatment from doctors within the healthcare centres, thus affecting their journey to health recovery (R14 and R17).

The study results revealed that some patients received their most helpful experiences at the hospital, despite being diagnosed late, when the disease was at the advanced stage, thereby making the treatment interventions prone to complications, especially for patients with pre-existing medical conditions (R10, R12 and R14).

Additionally, one of the participants echoed the concerns about the lack of continued linkage to care once they exit the health facilities (R14 and R16). Furthermore, the study offered a broad perspective on recognising patterns in the treatment-seeking behaviour and fears emanating from the perception of contagion (being re-infected, daily survival food and access to money). For instance, R10, a patient diagnosed with eye cancer who had previously been in remission, but relapsed had this to say during the support group meeting:

‘I wouldn’t lie, we get all the attention and care we need at the hospital facilities. But once, I get discharged it is a problem. My wife ran away after my diagnosis, so I have my aging mother to take care of me. It is difficult. Social workers do come; however, they stand by the gate with porridge and sometimes bandages. However, I am under major stress, we are struggling financially. Emotional help would be much appreciated, please let us know if you know someone.’ (R10, support group member, unemployed, 11 February 2019)

### Stigma and disclosure complexities

Sharing emotional and physical suffering with family members necessitated that the family member living with cancer disclosed his or her health condition. Participants reported how they grappled with negotiating disclosure of the diagnosis, as well as receiving support in making decisions on treatment options. For instance, in the conversation with two patients, this is what transpired:

‘My friends all got shocked, that is the hardest part actually, having to tell people that you are not well, you know because you look healthy, you look fine, basically physically nothing is wrong with you, when you are looking at me but inside something is wrong.’ (R8, support group member, runs a community crèche, 06 December 2018)

Similarly, R5 revealed that it was very difficult to disclose her cancer status to her family members. She opted to only disclose it to her older son as opposed to the whole family as she felt that this was a more sensitive topic.

The interviews elicited disclosure stories that were common to the one shared by R12. Notably, patients were worried about not being able to disclose on their own terms because of the risks associated with it, including anxiety and fear. Unpredictability to the disclosure outcomes was what scared patients living with cancer most. Most patients who had disclosed their tuberculosis (TB) or HIV and AIDS status employed distinctive, premeditated strategies. As in the case of R12, she was too afraid to disclose her cancer status to her family and publicly, fearing for potential reactions from her children, but instead, she opted to disclose her HIV status, which was relatively easy:

‘My children are under the assumption that I am attending a HIV support group. They are not aware that this is a Cancer support group as I haven’t disclosed to them. So, all that I am disclosing here, I am disclosing for the first time.’ (R12, support group member, group secretary, 12 February 2019)

Stigma was a common experience after being diagnosed with cancer, and this became a common threat reported by all cancer patients participating in the study.

Other support group members substantiated their concerns and shared that stigma limited their freedom of mobility. Clearly, cancer seemed tainted with a reputation of death. The inability to express feelings and concerns about their experiences triggered negative reactions in people, resulting in patients withdrawing from work or leisure activities, as expressed by R9:

‘I get angry now, I don’t like people to feel pity for me because I have one breast, I do not like that at all. I even changed the place where I receive the grant money as I wanted to run away from people feeling pity for me as much as I can. I hardly go out now, even when a relative of mine is getting married, I will only go and hand in the present and come back.’ (R9, active support group member, pensioner, 11 February 2019)

Evidently, an altered body, which is perceived as a visible sign of dying, caused distress and affected participants’ well-being and relationships with others. As stated earlier in the conversation, the inability to express their feelings and concerns for fear of negative reactions from people resulted in patients withdrawing from work or leisure activities. Consequently, this made the experience for patients a lonely one. Most patients were cautious of not becoming a burden to their families, thus resorting to hiding their distress. The effects of treatment and symptoms further affected their ability to communicate with others, which, in turn, made them feel isolated and not fully functional in their communities and society at large.

### Rationalising the illness through the lenses of faith and spirituality

This study found that whilst patients with the different types of cancer rationalised their illness differently, they all used faith and spirituality as the basis for their rationalisation:

I believe that I was given this disease by God as you know that people have different diseases. I believe that there is no disease that does not bring misery, if you have a sickness, you are not supposed to feel well anyway.’ (R10, support group member, unemployed, 11 February 2019)

R5 expounds on this statement:

‘I don’t want to lie, but I believe that everything happens for a reason, I believe that this too is God’s plan. God had planned long time ago that I will be killed by cancer.’ (R5, cancer patient and support group member, 29 November 2018)

In as much as R8 was accepting defeat, however, the plight of the disease rested with God in her case. In hearing this, the researcher then had a follow-up question asking if she believed that she was going to get healed, to which she responded:

‘I believe that I am going to get healed because I have a sister who has cancer, she usually called me and told me about her cancer journey.’ (R5, cancer patient and support group member, 29 November 2018)

Evidently, spirituality influenced help-seeking behaviour. Most participants viewed spiritual care as a form of self-care:^15^

‘I even tell God, “You know what? I am prepared for death because I don’t want to be a burden or be in the last minute of suffering”.’ (R1, palliative care patient, unemployed, 29 November 2018)

Spirituality helped such patients from the support group to deal with stress and uncertainty following a primary diagnosis of cancer. This was reiterated by R13 when she stated:

‘I am committed to the Lord, I surrendered my whole life to him, I said come what may, I will not go to anybody who will see healing and all that. When I was sick, only the church used to come and pray for me at home.’ (R13, pensioner, voluntary social worker, 19 February 2019)

For some participants, the view of cancer as a killer contributed to cancer patients viewing God as the only source of power to fight the disease rather than unquestioningly accepting biomedical treatment. For example, R1 asserted:

‘Yes, I always tell them that if you are failed by God, you must know there is nothing the doctors can do to help you. If the doctors are failing, we must continue praying till our final days.’ (R1, palliative care patient, unemployed, 29 November 2018)

‘Sometimes, I will wake up at 12:30 at night, I would be sitting and praying. I would say, “Lord there is no other God, you said you are healer and divine, you are going to heal me”.’ (R13, pensioner, voluntary social worker, 19 February 2019)

And he healed you from cancer? The Interviewer asked:

‘Yes, on the 5th of September when the doctor gave me that letter saying everything is normal, but now it is just that lump!’ (R13, pensioner, voluntary social worker, 19 February 2019)

Patients also shared their experiences of encounters with healthcare workers who instigated the belief that their power in healing lies in the power of God. For example, R14, a patient who attended YX Hospital mentioned that doctors would often say:

‘*Yebo siyanisiza, kodwa nathi sicela nisithandazele, nisibeke emthandazweni yenu*’. [Of course, we are here to provide help, but please pray for our strength as well]. Even though these religious beliefs had a positive effect, especially in meeting psychosocial needs, they however contribute to delays in seeking help.’ (R14, active support group member, 06 December 2018)

## Discussion

The objective of this study was to explore the experiences of patients living with cancer in support group settings in KwaZulu-Natal, South Africa. This study revealed that for patients living with cancer, diagnosis is not just a medical process, but it speaks about complex issues of how they engage with their families, society and social circles, as well as about matters of faith and spirituality.^16^ From these study findings, it was clear that the diagnosis of cancer also comes with diverse challenges, especially during and after treatment. These challenges include slow health improvements, recurrent or new emotional and physical challenges, and distress associated with procedures, a phenomenon that is pervasive in other studies.^17^ Cancer survivors of all ages are likely to experience common life disruptions secondary to cancer, and these include goal interference, altered interpersonal relationships and body image.^17^ Likewise, Meyerowitz, Kurita and D’ Orazio^18^ have also asserted in their study that survivors frequently have circumscribed areas of persistent difficulties, including fears of recurrence and psychological reactions to the physical dysfunction and disfigurement caused by some treatments.^18^ Similarly, problems, such as stress, long waiting times and distance between patients’ homes and healthcare facilities, are part of the challenges faced by patients living with cancer. For instance, a study conducted by Ambroggi, Biasini, Giovane, Formari and Covanna^19^ confirmed that ‘[d]istance from the hospital, or travel burden, had a negative impact on patients affected by cancer, in terms of the stage at diagnosis, appropriate treatment received and prognosis’.^19^ Similarly, the receipt of timely attention is central to high-quality care, in that delays in receiving care may lead to a more advanced disease and subsequently poor treatment outcomes.^20^ Social issues such as disclosure and stigma were also found to play a big role in how patients experienced their illness.^21^ For instance, in studies on patients from Ethiopia and Uganda, public stigma associated with breast cancer resulted in internalised stigmatisation, which delayed care engagement.^21^

This is one of the few ethnographic studies that explored experiences of patients with cancer, by combining together a wide range of personal and individual experiences of the affected people. Through an ethnographic approach, this study developed new ways of understanding patients’ illness.

In this study, it was evident that cultural beliefs influenced individual’s health-seeking behaviours. Surbone^22^ elaborated that cultural groups have systems of health beliefs to explain what causes illness, how can it be cured or treated and who should be involved in the process.^22^ The study reiterated the presence of cultural construction of cancer illness, specifically through framing the religious influence in health-seeking behaviours. Evidently there were various cultural practices and beliefs practised by cancer patients as a way of dealing with the disease. Likewise, through culture, individuals relate, communicate and connect with each other to make sense of the world.^22^ Additionally, families brought up culture-specific ideas and values related to concepts of health and illnesses.^23^ This shaped the way patients understood their illness and the measures they took to overcome their illness.

Structural factors related to healthcare services, such as long process of admission, and challenges with referral systems resulted in patients experiencing frustration and fatigue when following their treatment.^15^ The results of this study are reflective of the South Africa’s struggling healthcare system.^6^ In the recent years, issues such as the shortage of machines have been raised and are yet to be fully addressed.^6^ Cancer affects the emotional standing of patients, and hence, there is a need for psychosocial support outside the hospital setting.

This is deemed as one of the influences to delay in seeking treatment, apart from the documented financial challenges.^24^ Having a chronic illness causes disorder to an individual’s normal daily routine and can cause behavioural disruption, affecting how patients perceive themselves and or their belief about how others perceive themselves.^24^ Evidently, the experience of illness is not bounded by the bodies or consciousness of those who are ill, rather, it reaches out to encompass the household, a family, society or a social network, and runs deep into the inner worlds of patients, yet decidedly transpersonal.^16^ Although there was considerable variation in the detail of how each patient was diagnosed and treated, the family was also affected. Whilst HIV and AIDS has come a long way in the fight against stigmatisation of an illness, cancer appears to be on a similar trajectory, as patients living undergo stigmatising experiences. Although stigma experienced in HIV and AIDS and cancer patients may not necessarily be the same, there are considerable overlaps given the large co-infection rate in South Africa.^25^

### Strength and limitations

The strength of this study lies in the fact that it was conducted using an ethnographic design, which captured the participants’ experiences in their own natural setting. The researcher spent months in the support group setting, which helped in building rapport, necessary for obtaining rich data.

The data from study participants were obtained from one province. However, the detailed exposition of the research process may make the study findings transferable to the other support groups with the comparable context elsewhere in the country. Even though, support groups are well recognised, however, they attracted a very small proportion of people diagnosed with cancer. Furthermore, revolving around different sites proved to be difficult, as it meant that a limited time was spent by the researcher on each site.

Furthermore, the conversations were sometimes brief and not well articulated because of the condition of patients. The average age of the patients was quite high, and there were relatively few men, thereby limiting the diversity of perspectives from men and younger patients.

### Implications or recommendations

The results of this study make a case for each patient’s suffering to be viewed within the context of a wider spectrum of adversity. In addition, research methodologies, such as ethnography, offer deep emotional account of patients’ experiences at a very personal level than would be the case with other methodologies. The results of this study may be used to design interventions aimed at offering support for patients living with cancer in order to cope better with their illness. Future research should examine the experience of social support in those who do not attend support group settings to provide insights and contrast between family social support and support from other sources.

### Contribution of the evidence-based research

From a public health and an anthropological point of view, this study offers an important contribution to a body of literature on the understanding of patients’ experience of living with cancer in support group settings. The value of this research outside the public health sector lies in its ability to bring the complex meanings, given the experience of cancer and the cultural construction of illness in how patients rationalise their illnesses.

Most ethnographic studies have been undertaken in Western, high-income countries, as opposed to non-Western countries, whereas there is still a scarcity of in-depth ethnographic studies on the social meanings and dimensions of health, and therapeutic practices in modern hospitals. As a result, this study hopes to make contribution to the qualitative literature on the experiences of illness and hospital life in South Africa. This could also be used as a model for other African countries.

## Conclusion

This ethnographic study has demonstrated the complexity of issues faced by patients when diagnosed with cancer within a support group setting, including financial constraints, high cost of healthcare, previous negative experience with healthcare workers, reinforced poor treatment and delayed treatment for patients. Patients perceived support groups to be playing an important role in improving their understanding of the cancer prognosis, as well as in a better management of the disease. Ethnographic perspectives provide complimentary insights into those that would be gained using more traditional qualitative research. Further research is warranted to extend this analysis across the wider trajectory of chronic illnesses and to a variety of disease types. Education should certainly be the starting point, but by no means enough to improve the quality of life for citizens. Notwithstanding, this ethnographic study can be considered a first step towards discovering how recent developments in cancer experience will shape clinical and research domains in the management of cancer and other chronic diseases. By providing insights into the experiences of patients living with cancer and social dynamics of healthcare, this study contributes to increasing awareness about the current developments in the care of patients with different types of cancers.
